# Mapping Cold-Water Coral Habitats at Different Scales within the Northern Ionian Sea (Central Mediterranean): An Assessment of Coral Coverage and Associated Vulnerability

**DOI:** 10.1371/journal.pone.0087108

**Published:** 2014-01-23

**Authors:** Alessandra Savini, Agostina Vertino, Fabio Marchese, Lydia Beuck, André Freiwald

**Affiliations:** 1 Milano-Bicocca University, Dept. of Geological Sciences and Geotechnologies, Milano, Italy; 2 Senckenberg am Meer, Marine Research Department, Südstrand, Wilhelmshaven, Germany; Heriot-Watt University, United Kingdom

## Abstract

In this study, we mapped the distribution of Cold-Water Coral (CWC) habitats on the northern Ionian Margin (Mediterranean Sea), with an emphasis on assessing coral coverage at various spatial scales over an area of 2,000 km^2^ between 120 and 1,400 m of water depth. Our work made use of a set of data obtained from ship-based research surveys. Multi-scale seafloor mapping data, video inspections, and previous results from sediment samples were integrated and analyzed using Geographic Information System (GIS)-based tools. Results obtained from the application of spatial and textural analytical techniques to acoustic meso-scale maps (i.e. a Digital Terrain Model (DTM) of the seafloor at a 40 m grid cell size and associated terrain parameters) and large-scale maps (i.e. Side-Scan Sonar (SSS) mosaics of 1 m in resolution ground-truthed using underwater video observations) were integrated and revealed that, at the meso-scale level, the main morphological pattern (i.e. the aggregation of mound-like features) associated with CWC habitat occurrences was widespread over a total area of 600 km^2^. Single coral mounds were isolated from the DTM and represented the geomorphic proxies used to model coral distributions within the investigated area. Coral mounds spanned a total area of 68 km^2^ where different coral facies (characterized using video analyses and mapped on SSS mosaics) represent the dominant macro-habitat. We also mapped and classified anthropogenic threats that were identifiable within the examined videos, and, here, discuss their relationship to the mapped distribution of coral habitats and mounds. The combined results (from multi-scale habitat mapping and observations of the distribution of anthropogenic threats) provide the first quantitative assessment of CWC coverage for a Mediterranean province and document the relevant role of seafloor geomorphology in influencing habitat vulnerability to different types of human pressures.

## Introduction

Cold-Water Coral (CWC) habitats form one of the most Vulnerable Marine Ecosystems (VME) to human pressures within the deep sea [1]–[3]. CWCs are especially vulnerable to damage by the mechanical impacts of bottom fishing activities [4]–[11]. Only by exception, due to the extreme seafloor complexity of the physical environment in which they occur [12], [13], is the impact by bottom trawling minimal or absent. With the rise of human activity within the deep sea, such habitats are under increasing pressure from a number of different [14], [15], synergetic [3], and cumulative threats [16] (e.g. pollution and litter, aggregate mining, oil and gas exploration, coastal development, cables, shipping, invasive species, climate change, etc.). Many of these threats are common to all deep-sea habitats and fauna although it is still not clear how a given deep-sea habitat can be differentially impacted by each, and how their synergetic impacts vary according to habitat structure.

To efficiently combine human use of marine resources (responsible for the main threats) with associated ecosystem functions (as broadly discussed, among others, by [17]), and to foster the responsible and conscious management and conservation of offshore resources, the production of benthic habitat maps at a variety of spatial scales is required [18]–[20]. European research (for example, MESH (http://www.searchmesh.net/), MAREANO (http://www.mareano.no/), EU-FP6 HERMES (http://www.eu-hermes.net/), EU-FP7 HERMIONE (http://www.eu-hermione.net/), and EU-FP7 CoralFISH (http://www.eu-fp7-coralfish.net/)) and government-supported projects have recently included suitable benthic habitat maps for Atlantic waters (e.g. [21] and [22]) as a basic tool for supporting the implementation of European and national legislation such as Habitats and Marine Strategy Framework Directives, as well as new marine management initiatives (e.g., Marine Spatial Planning (MSP) and Ecosystem-Based Management (EBM) [23]–[25]).

For the deep Mediterranean Sea that contains 7% of the total marine biodiversity of our planet [26], and where the slope and, in particular, deep basins cover a major portion of the entire basin (over 70%), accurate maps representing the distribution of deep-sea benthic fauna are absent [27]. Only recently have focused research programs (e.g. APLABES, [28], EU-FP6 HERMES, EU-FP7 HERMIONE, and EU-FP7 CoralFISH) promoted international oceanographic expeditions using the latest generation of acoustic devices, Remotely Operated Vehicles (ROVs), and manned submersibles in order to systematically explore the Mediterranean Sea’s deep seabed. As a result of such programs, the occurrence of VMEs has been revealed (e.g. CWC habitats or seep-related seafloor habitats) in several Mediterranean Sea deep-sea settings, as follows: in the western Mediterranean Sea (the Alboran Sea, [29], the Cape de Creus Canyon [30], [31], and the Gulf of Lion [32]); in the Sicily Channel [33]–[35]; in the Ionian Sea [28], [34], [36], [37]; and in the southern Adriatic Sea [34], [38]. Most of these deep VMEs have not yet been mapped, and, as a result, a comprehensive geomorphological and ecological characterization of Mediterranean Sea deep-sea habitats is still lacking.

According to the marine habitat classification concepts for ecological data management [39], [40], most past geomorphological and ecological research performed within the Mediterranean Sea deep environment has been conducted at the *global/mega-scale* (i.e. a spatial scale of 100–10 km) and at the *nano/micro-scale* (i.e. a spatial scale less than 1 m). Only recently has the *meso-scale* (i.e. a spatial scale of 10–1,000 m) received attention, due to the production of benthic habitat maps [30] [41], although environmental management plans (such as the ones of the Marine Strategy Framework Directive (MSFD) that still needs to be established) require reliable knowledge of seafloor features at the meso-scale level (sometimes referred to as the “*seascape*” scale of the marine environment [39], [40]). Investigations of deep-sea habitats using videos have indicated that, for most cases, the distribution of fauna exhibits patterns of variability on spatial scales smaller than the resolution that most common ship-borne acoustic devices are able to image and map for the seafloor (i.e. MultiBeam Echo-Sounder (MBES) systems) [42]. However, such investigations have also revealed that certain fauna (such as CWC) can exhibit tendencies that are associated with larger scale features of the terrain, such as mounds [43], suggesting that larger scale features may represent important contributors to the distribution of some fauna, especially benthic fauna that exhibit a “preference” for particular types of terrain. Terrain variables and environmental factors (depth, slope, sediment properties, water mass properties, hydrodynamic regime, etc.) have typically been used to perform predictive distribution models for mapping different biota within various marine settings (see [44] and references therein for a complete list of the various types of predictive models). Since environmental variables are generally much more readily available and easier to obtain than biological observations, the inductive approach of these models is very useful. Numerous types of predictive models have been employed for mapping habitat distributions [44], [45]. In particular, Habitat Suitability Modelling (HSM) has proved to be a valuable tool for estimating deep-sea coral distributions on a global scale [46]–[48].

To estimate coral coverage at a variety of spatial scales, our work sought to map the distribution of CWC habitats within the northern Ionian Sea using a quantitative approach. We examined ways in which we could build outward from video surveys to model (i.e. predict) coral distributions and coverage (beyond the field of view of a underwater camera) to determine a multi-scale visualization of the study environment. To extrapolate the most relevant morphometric and acoustic features associated with the occurrence of CWC habitats, acoustic data collected by a MBES and a high-frequency Side-Scan Sonar (SSS) were analyzed using a suite of quantitative techniques. To groundtruth acoustic data and to add information regarding the occurrence of anthropogenic threats (i.e. litter, trawl-marks, and remnants of fishing gear), we combined results from the video analyses. The obtained multi-scale representation of CWC coverage within the northern Ionian Sea and the associated relationship with identified anthropogenic threats allowed us to provide a first insight into the vulnerability of CWC habitats within the study area.

### Study Area

**Figure 1:**
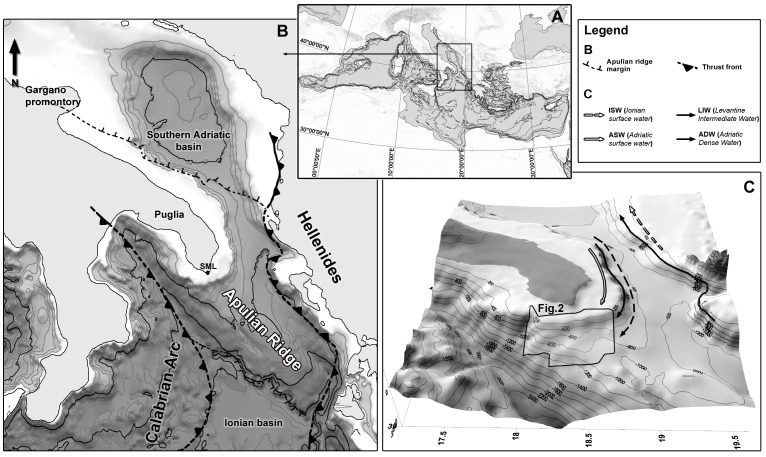
The geographic, geomorphological, and oceanographic framework of the study area (modified from [50]). A: The geographic framework of the study area within the Mediterranean Sea. B: The geological setting showing the Apulian Ridge as the foreland system of both the Appennines and Hellenic fold-and-thrust belts. B: The oceanographic setting showing ASW (Adriatic Surface Water), LIW (Levantine Intermediate Water), and ADW (Adriatic Dense Water) within the study area.

The study area is located within the northern Ionian Margin (eastern Mediterranean Sea) at the southern prolongation of the Apulian Peninsula (south-eastern Italy) in the Ionian Sea, along the Apulian Ridge (Figure 1a, b). The ridge extends from Apulia to offshore Greece and separates the southern Adriatic Basin, at the southern edge of the Otranto Channel, from the deeper Ionian Sea; and is a part of the present foreland system of both the Apennine Arc to the west and the Hellenic Arc to the east [49]. Recent high-resolution bathymetric and shallow seismic surveys [50] have revealed that the NNW-SSE normal fault network, which crosscuts the seabed, results in a number of prominent fault scarps and promontories that control the large-scale morphology of the margin [51]. Overall, sedimentation is basically characterized by mass-wasting deposits, likely associated with the local high seismicity of the margin, often regarded as a consequence of the supposed activity of the NNW-SSE normal fault network of the Apulian Ridge [49], [52], [53]. Evidence of mass movements for the main sedimentation process within the margin is provided by the presence of large arcuate headscarps, indenting the shelf break and the superficial deformation (i.e. compressional and extensional ridges, low scarps, and lineations) of mass-failed deposits located within the upper slope, characterized by a noteworthy blocky pattern that extends over more than 600 km^2^ [50]. Furthermore, as documented by the occurrence of sediment drifting [36], [50], the margin is also impacted by bottom currents.

The northern Ionian Sea receives water from the southern Adriatic Basin. In the study area, a core of cold ( = 12.92°C), less saline (38.64%), and oxygenated water of Adriatic origin flows from the Otranto Channel and moves in geostrophic balance along isobaths of 600–1,000 m in depth (Figure 1c) [54]. During its flow toward the Ionian Sea interior, Adriatic Dense Water (ADW) mixes with bottom water, changes thermohaline properties, and becomes Eastern Mediterranean Deep Water (EMDW) [54], [55]. At the same depth range, living and sub-modern *Madrepora*-dominated coral communities have recently been identified within the study area and are referred to as the “Santa Maria di Leuca (SML) CWC province” [28], [56]. The corals display a patched distribution across a wide sector of the margin and are associated with the blocky pattern of mass-failed deposits, capping clustered or isolated mounds, 50–300 s in diameter and up to 25 m in height [50]. Some of these morphological features have been interpreted as “coral mounds” [41] and appear in seismic data as very high acoustic transparent hyperboles (echotype III_1, III_2, and III_3 in [50]).

## Data and Methods

**Figure 2:**
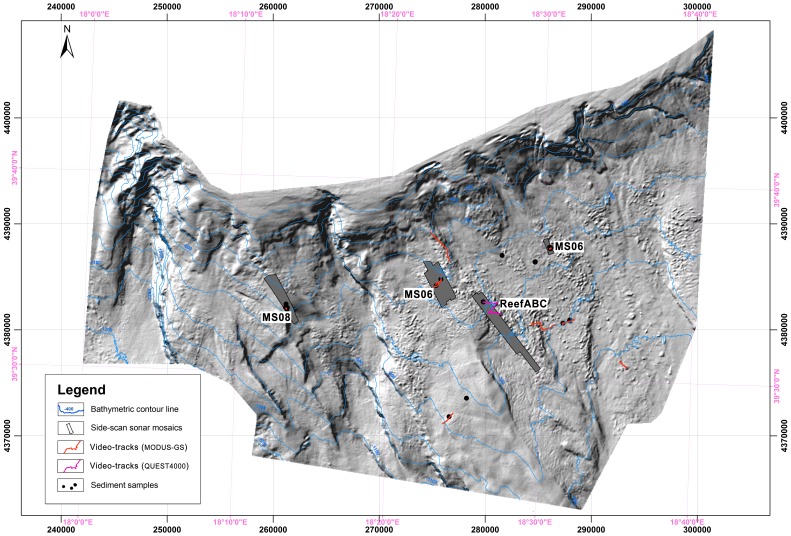
A shaded relief map (40 m grid – artificial sun angle from NNW – vertical exaggeration five) provided by MBES surveys carried out from 2004 (APLABES cruises) to 2010 (Magic-CoralFISH cruise); the contour spacing is 100 m. Polygons indicate the areas covered by SSS mapping. Dots indicate the sediment sampling stations (from [61], [62] and [70]). Red and pink thick track lines indicate, respectively, the towed camera and ROV dives performed within the area (from [50] and [34]).

We utilized a set of data obtained from five, ship-based research surveys (performed between July 2004 and April 2010). Acoustic data collected at various spatial scales, and video footage obtained from different ROV dives and from an underwater module equipped with cameras represented the core of the data set (Figure 2). The acoustic data set was composed of seismic profiles, high-quality bathymetry data, and high-frequency SSS mosaics. Video data were acquired using two different underwater video-recording systems - the tethered MODUS, GAS-SCIPACK (MGS) instrumentation [41], [50], [57], and the MARUM ROV ‘QUEST 4000’ [34]. To represent the distribution of CWC habitats using a quantitative approach and to estimate CWC seafloor coverage at a variety of spatial scales, all data were integrated and analyzed using GIS-based procedures (using ArcGIS™ software).

### Bathymetry: Data Origin and Analysis

Bathymetric data were acquired using a Reson Seabat 8160 MBES. In total, a 2,000 km^2^ survey area was covered by multibeam data (Figure 2) with a 20/40 m resolution (depending on the surveyed depth) collected during two main oceanographic cruises (the 2004 APLABES cruise and the 2010 MAGIC-CoralFISH cruise). Data were processed using dedicated software (Caris Hips and Sips 6.1) to produce a Digital Terrain Model (DTM) of the entire survey area that extended from 120 m in water depth (w.d.) on the continental shelf down to 1,400 m w.d. on the slope (Figure 2). The survey area was represented by a DTM (raster data set) with a cell size of 40×40 m (Figure 2). Terrain Morphometric Attributes (TMAs) were extracted from the DTM. TMAs represented the raster data set where each pixel (*x*0, *y*0) was assigned a value calculated from the *z* values of the DTM. TMAs (*derivates*) were generally grouped by slope, aspect, and topographic curvatures. Six of the main TMAs were computed by applying topographic modelling using the ArcGIS™ spatial analyst extension’s surface analysis and the Landserf 2.2® software (used to compute the TMAs proposed by [58]). An additional algorithm, the Bathymetric Position Index (BPI), was also run in ArcGIS™ using the ArcGIS™ Benthic-Terrain Modeller extension. The slope, the orientation (aspect), and the curvatures were computed using a window analysis of 3×3; whereas the BPI was calculated over a range of scales (Table 1). Based on [59] and [60], maps generated by all of the provided TMAs were used to produce descriptive maps that helped to identify areas with a set of different morphometric properties that may be linked to typical seafloor geomorphologies. Following [60], we subsequently analyzed surface roughness by calculating the moment statistics of all of the computed TMAs. The standard deviations (SD) from all of the curvature maps and the mean slope were calculated. We then processed different RGB images by using the computed maps that represented TMAs and their moment statistics as input bands.

**Table 1:** Terrain variables derived from multibeam bathymetry data (DTM).

Type of Terrain Analysis		Analysis window	Software
Slope	Slope	Rectangle of size *n*: 3	ArcGIS™; Landserf 2.2
Orientation	Aspect	Rectangle of size *n*: 3	ArcGIS™; Landserf 2.2
Curvature	Profile curvature	Rectangle of size *n*: 3	ArcGIS™
	Plan curvature	Rectangle of size *n*: 3	ArcGIS™
	Cross-sectional curvature	Rectangle of size *n*: 3	Landserf 2.2
	Longitudinal curvature	Rectangle of size *n*: 3	Landserf 2.2
	Minimum curvature	Rectangle of size *n*: 3	Landserf 2.2
	Maximum curvature	Rectangle of size *n*: 3	Landserf 2.2
	Mean curvature	Rectangle of size *n*: 3	Landserf 2.2
	Bathymetric Positioning index (BPI)	Circle of radius *n*: 1, 5, 7, 10	ArcGIS™
Moment Statistics	Standard Deviation (SD) of Slope	Rectangle of size *n*: 3	ArcGIS™
	Slope average	Rectangle of size *n*: 3	ArcGIS™
	plan curvature SD	Rectangle of size *n*: 3	ArcGIS™
	profile curvature SD	Rectangle of size *n*: 3	ArcGIS™
	BPI SD	Circle of radius *n*: 1, 5, 7, 10	ArcGIS™
	profile	Rectangle of size *n*: 3	ArcGIS™
	plan curvature	Rectangle of size *n*: 3	ArcGIS™
	crossectional SD	Rectangle of size *n*: 3	ArcGIS™
	longitudinal curvature SD	Rectangle of size *n*: 3	ArcGIS™
	maximum curvature SD	Rectangle of size *n*: 3	ArcGIS™
	minimum curvature SD	Rectangle of size *n*: 3	ArcGIS™
	mean curvature SD	Rectangle of size *n*: 3	ArcGIS™

The value *n* or *r* defines the number of raster cells included in the analysis window surrounding the central pixel, and determines the scale of the terrain analysis. Selected values from three (local scale) were utilized for all computations, with the exception of the Bathymetric Positioning Index, for which we performed a multi-scale computation.

We further used a suite of GIS-based tools (i.e. the Isocluster Unsupervised Classification that combines the Iso-Cluster and Maximum Likelihood Classification - MLC) to extract and classify all of the mound-like morphologies as distinct polygons from the DTM and to perform an additional analysis in order to localize suitable morphologies for CWC colonization. From all of the isolated polygons, those occurring in a depth range located between 400 and 1,000 m in w.d. (the same depth range of coral occurrences in our study area – [54]) and those represented on the seismic profiles by a chaotic pattern exposed at the seafloor (i.e.: echotype III_1, III_2 and III_3 of [50]) were selected.

### SSS Imagery: Data Origin and Analysis

**Figure 3:**
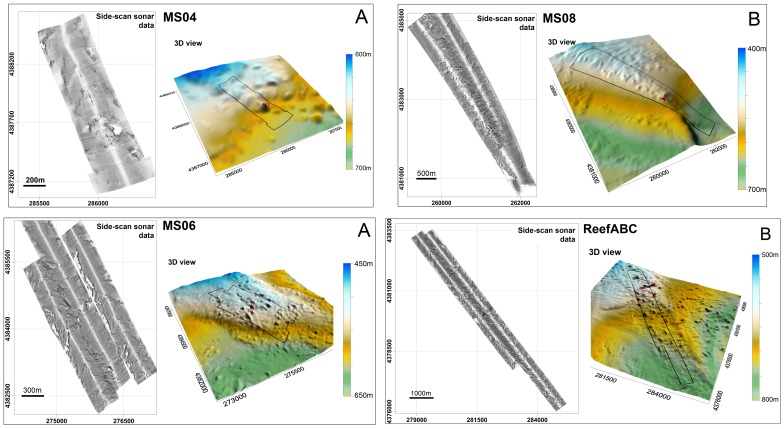
A: The SSS mosaic (left) and the equivalent 3D from the DTM (right) at site MS04, see Figure 2 for the location. B: The SSS mosaic (left) and the equivalent 3D from the DTM (right) at site MS08, see Figure 2 for the location. C: The SSS mosaic (left) and the equivalent 3D from the DTM (right) at site MS06, see Figure 2 for the location. D: The SSS mosaic (left) and the equivalent 3D from DTM (right) at site ReefABC, see Figure 2 for the location.

**Figure 4:**
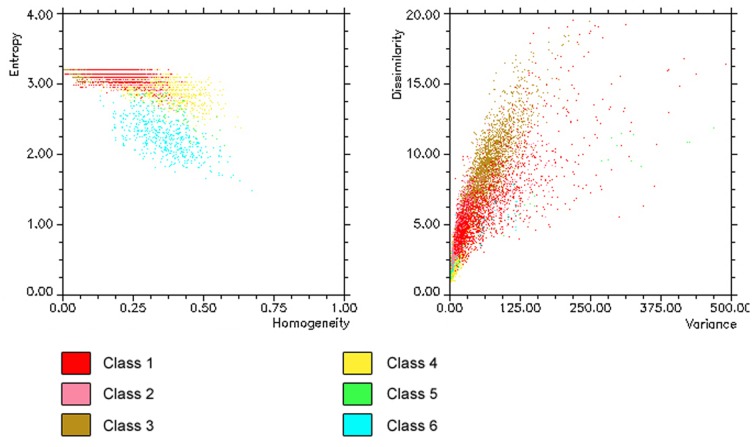
Scatter plots displaying the values of entropy and homogeneity (left), and the dissimilarity and the variance (right) for the acoustic facies listed in Table 3.

Exploration by means of a 100–500 kHz SSS (Klein 3000) was performed in an operating range of 300 m at four main sites (MS04; MS06; Reef ABC; MS08 - Figure 2) located between 400 and 700 m in w.d. over an area shaped by a blocky pattern [50] where CWC occurrences were documented through video surveys and the collection of sediment samples ([34], [41], [61], [62]). SSS data processing, performed using Triton Elics Information (TEI) suite software packages, produced geo-referenced gray-tone acoustic images of the seafloor at a 1 m resolution (Figure 3). The DTM provided by multibeam surveys was used for the final georectification of processed SSS mosaics. We computed quantitative textural measurements on SSS mosaics using an application of the co-occurrence matrix [63]. Our analysis was based on the theoretical work by Haralick [63]; and practical applications to sonar imagery reported by several authors (among others [64]–[68]) who have indicated that co-occurrence matrices are the most adapted tools for quantifying and outlining textures from SSS imagery. Co-occurrence matrices are represented by statistical measures called indices. Using ENVI4.3® software and following Huvenne et al. [67], we computed entropy and textural homogeneity (two indices). According to Savini [68], another four indices were added to the analysis, as follows: the mean, the variance, the dissimilarity, and the second moment. The dimension of the matrix applied to the data input, which defines the *cell size* at which the output raster is created, was 5×5 pixels. Six distinct types of backscattering (i.e. SSS acoustic facies) were defined according to their textures (qualitative description – Table 2). Differentiation of the six acoustic classes, produced by their textural proprieties, was investigated by analyzing the range values for all six of the computed indices (Figure 4). We further performed a supervised classification by applying the MLC algorithm (using ENVI4.3® software) over the six computed textural indices. Such a classification type requires the selection of training areas for use as the basis for the classification. Our training areas were the six acoustic facies (Table 2). Each class was then converted into polygons (following integration of the classification maps in ArcGIS™) in order to quantify the total coverage of each acoustic facies within each mosaic.

**Table 2:** A qualitative description of backscatter textures.

SSS *Facies*	Description
Class 1	Speckled pattern of intermediate to very high backscatter
Class 2	Patchy pattern of intermediate to high backscatter
Class 3	Homogenous pattern of low backscatter
Class 4	Homogenous pattern of intermediate backscatter
Class 5	Homogenous pattern of very high backscatter
Class 6	Homogenous pattern of very low backscatter

We also investigated the correlation between the seafloor distributions of the six classes, with results obtained from video analysis (i.e. ground-truthing). Our interpretation is then discussed following a presentation of the results from the video analysis (see discussion hereafter) and an accuracy assessment of the provided SSS classification maps performed according to Pontius and Millones [69]. To calculate omission and commission, including agreement for each acoustic class (as specified by [69]), we used the sample matrix (Table S1) derived from the correlation matrix obtained between interpreted ROV data at the ReefABC site (that represents the most accurate georeferenced video data we have for the investigated area) and the associated SSS classification map.

### Video Data: Origin and Analysis

Video data from sites MS04, MS06, and MS08 were collected during a cruise on the R/V *Universitatis* [51]. Videos were recorded by three cameras mounted on the MGS vehicle, and by the light workclass PLUTO 1000 ROV [41]. Three MGS video cameras were mounted on one side (‘lateral camera’), in front (‘front camera’), and at the base (‘bottom camera’) of the MGS module. The first two cameras were inclined at approximately 45° with respect to the horizontal plane; whereas the third camera was oriented vertically (technical details are provided in [41] and [51]). All of the collected videos were analyzed for macrofauna identification and for qualitative habitat characterization. The percentage of seafloor coverage, used to distinguish the 13 macrohabitats mentioned in the text (Table 3), was estimated by point counting on selected videoframes extracted from the bottom camera video transect. Due to variable speed of the MGS module, the video frames were captured at variable time intervals (generally from approximately 5 to 20 s) to avoid overlap between subsequent 1 m^2^ images and to obtain almost continuous video coverage of the seafloor. A standard window (“quadrat”), corresponding to a seafloor area of approximately 1 m^2^ and containing 100 equally-spaced points, was overlain on each extracted screenshot. Thanks to a fixed camera orientation (vertical) and the use of a 10 cm-long object as a reference for estimating the size of the standard quadrat, point counting on selected videoframes was possible. The object was attached to the base of the MGS frame and dragged along the seafloor during video recording (also see [41]). The survey track of the MGS bottom camera was extrapolated from the offset position of its cable at sea in relation to the DGPS antenna.

**Table 3:** Video-detected macrohabitats and the percentages of their main components.

HABITAT	coral colonies	coralrudstone	coral rubble	mudstone	mud
**C (Coral framework)**	>75%				<25%
**Crd (Coral rudstone)**		>75%			<25%
**Ms (Mudstone)**				>75%	<25%
**Cr (Coral rubble)**			>75%		<25%
**M (Mud)**					c. 100%
**CM (Corals and Mud)**	25–75%				<75%
**MC (Mud and Corals)**	<25%				>75%
CrdM (Coral rudstone and Mud)*		25–75%			<75%
**MCrd (Mud and Coral rudstone)**		<25%			>75%
**CrM (Coral rubble and Mud)**			25–75%		<75%
**MCr (Mud and Coral rubble)**			<25%		>75%
MsM (Mudstone and Mud)*				25–75%	<75%
**MMs (Mud and Mudstone)**				<25%	>75%

The percentages are referred to as 1 m^2^. The word “coral” indicates colonial Scleractinian. Empty cells in the tables indicate the absence of the relative component or a very rare occurrence at a low cover percentage (<<5%). Mixed habitats, CrdM, MCrd, MsM, and MMs, can be characterized by continuous hardground crusts partly covered by mud or by boulder fields on a muddy bottom. * indicates habitats where “Antipatharian facies”, as mentioned in the text, have been found.

Videos of the “Reef ABC” were recorded during the METEOR 70-1 cruise by the MARUM ROV ‘QUEST 4000’ [34]. QUEST uses a Doppler Velocity Log (DVL, 1200 kHz) to perform station-keep and displacement. Absolute GPS-based positioning was performed using the shipboard IXSEA POSIDONIA USBL positioning system, reaching absolute accuracy in the range of 5–10 m. Three cameras were mounted for the analysis - two color zoom cameras and a 3CCD HDTV video camera. Additionally, high-resolution digital still photographs obtained using a Nikon Coolpix camera were analyzed. The front-looking DSPL SEACAM 6500 was equipped with two laser points (with laser spacing of 20 cm), but the parameters (zoom, pan, and tilt) of this camera were not steady during video-recording. Therefore, seafloor coverage was not computed using point-counting. The “ReefABC” habitats were identified, following the classification presented in Table 3, by continuous video analysis of the front-looking camera. The visualized area size was estimated using laser points, when available, and/or known-sized objects (e.g. ROV robotic arm and frontal frame, the known size of organisms (e.g. the echinoderm *Cidaris*, anthropogenic objects etc.)).

In both cases (MGS and QUEST videos), all human impacts observed on the seafloor were recorded and counted, whether they were lost or discarded items or trawling traces on soft bottoms. In the statistical analysis carried out in this study, human threats were considered as variables and classified as disposal *(d)* (according to [3]) fishing line/net rests *(fl/n)*, and trawling traces *(t)*. Due to the different areal extension of the examined habitats (Table S2), the abundances of *d, fl/n* and *t* are expressed as number per 10 square meter in Table 4. To test the correlation between habitats and anthropogenic items/traces, the matrix shown in Table 4 (all sites) was imported into the PRIMER V6 software and analyzed using cluster, non-metric multidimensional scaling (MDS), and SIMPER methods. Cluster and MDS analyses were based on the Bray-Curtis (B–C) measure of similarity on square root-transformed data. The cluster analysis was displayed on a 2-D MDS plot by drawing slices through the dendrogram at fixed resemblance levels. To break down the contribution of each variable to the observed similarity (or dissimilarity) between and within habitat groups, the differences between identified groups of habitats were further investigated using the similarity percentages routine (SIMPER) (Table S3).

**Table 4:** A list of the abundance of anthropogenic items and the traces identified by video analysis at each macrohabitat.

		Crd	C	CM	MC	Cr	CrM	MCr	MsM	MMs	M
**MS04**	***d***	–	0.32	0.33	0.91	–	–	–	–	–	–
	***fl/n***	–	–	–	–	–	–	–	–	–	–
	***t***	–	–	–	–	–	–	0.06	–	–	0.07
**MS06**	***d***	0.12	0.98	2.08	0.79	–	0.15	–	–	–	–
	***fl/n***	0.24	–	0.6	4.19	1.3	0.86	0.26	0.5	0.48	–
	***t***	–	–	–	–	–	–	0.01	–	–	0.31
**MS08**	***d***	–	–	1.3	–	–	–	–	–	–	–
	***fl/n***	–	–	–	–	–	–	–	–	–	–
	***t***	–	–	–	–	–	–	–	–	–	–
**REEF"ABC"**	***d***	–	0.37	0.3	0.57	–	–	0.31	–	–	0.01
	***fl/n***	–	–	–	0.04	–	–	0.1	–	–	–
	***t***	–	–	–	0.11	–	–	–	–	–	–
**All sites**	***d***	0.12	0.43	0.41	0.62	–	0.12	0.08	–	–	0.01
	***fl/n***	0.24	–	0.04	0.59	1	0.69	0.19	0.24	0.41	–
	***t***	–	–	–	0.09	–	–	0.02	–	–	0.06

Abundance is reported for each site (MS04, MS06, MS08 and ReefABC) and for the total investigated area (all sites) expressed as the number of occurrences/10 m^2^. *d* indicates disposal (litter and solid waste, mostly plastic materials); *fl/n* indicates the rests of fishing lines and nets; and *t* indicates trawling traces.

### GIS Data Integration

**Figure 5:**
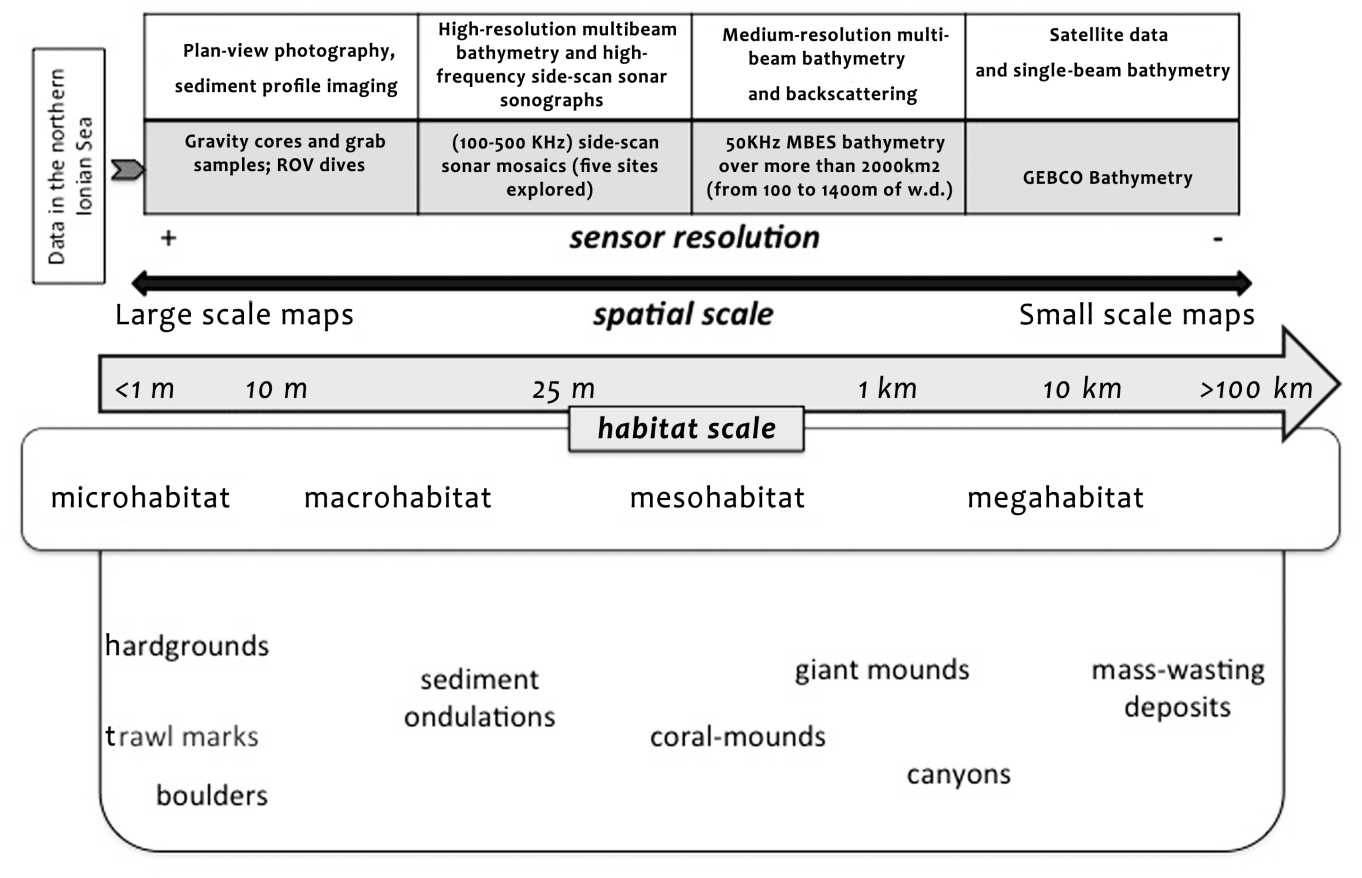
Some typical bathymetric features occurring within the study area, with an indication of their range of dimension. The indicated dimensions are related to the scale of the benthic habitats, the spatial scale of the maps in which they are represented, and the resolution of the sensors used to identify them on the seafloor. Habitat size classes were adapted from Greene et[39].

To perform CWC mapping on different scales, the DTM, the TMAs, the SSS mosaics, the classification maps, and all of the video tracks were integrated in ArcGIS™. According to Figure 5, different sources of data can be acquired in order to perform habitat characterization, and different resolution provides different spatial information as a result of habitat definition changes when the habitat distribution must be represented in maps. In our study, data resolution and associated map scales resulted, as follows:

1. The interpreted video tracks and seabed samples (data from previous work [34], [36], [41], [61], [62], [70]) provided invaluable insight into the biological and geological attributes of micro-scale habitat features (Figure 5);
2. The DTM (grid data – Figure 2) and the TMAs (raster data) had a spatial resolution (40×40m) that provided quantitative information that basically resolved meso-scale seafloor features using meso-scale maps (1∶50,000– Figure 5); and
3. SSS mosaics and the derived classification maps displayed a higher resolution than DTM, and resolved macro-scale seafloor features through large-scale maps (from 1∶1,000 to 1∶10,000, Figure 5).

## Results and Discussion

### Terrain Analysis and the Predictive Modelling of the Coral-mound Distribution

**Figure 6:**
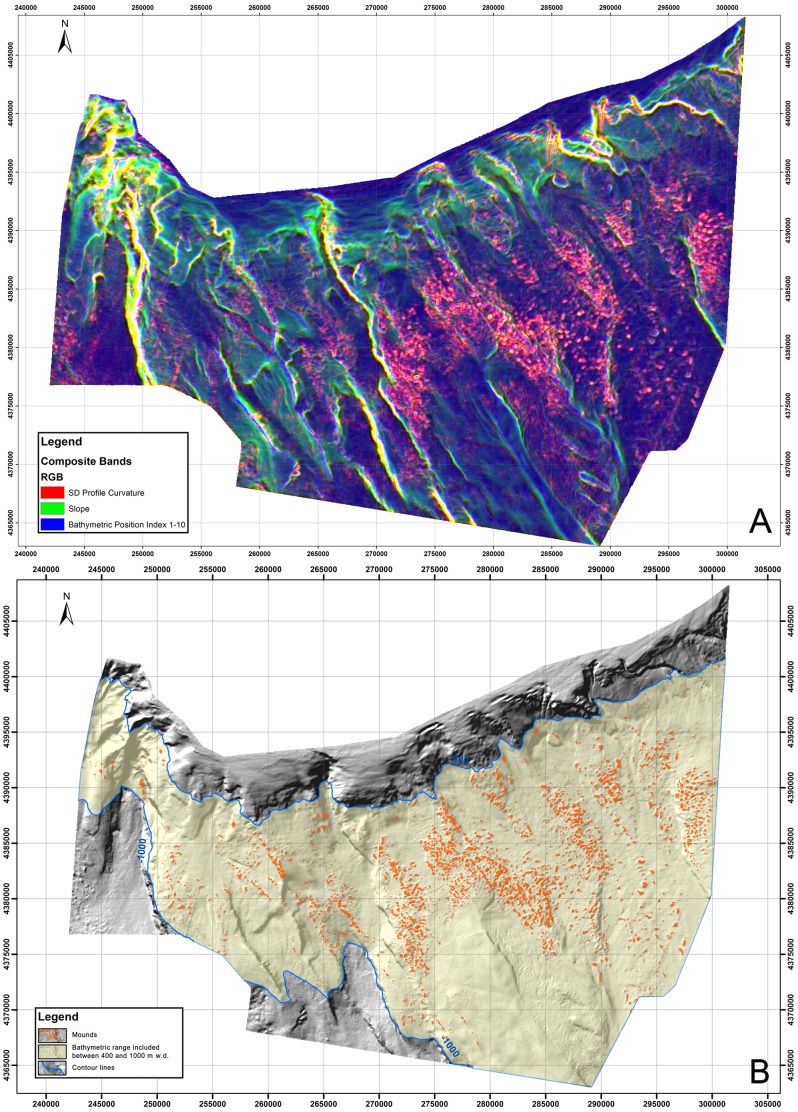
A: A RGB color composite image made by the following TMAs of the study DTM: the SD profile curvature (Red), the slope (Green), and the bathymetric position index 1–10 (Blue). B: The location of all of the extracted polygons that represent coral-mounds (in orange) on the study DTM. Light yellow areas highlight the sector included between 400 and 1000 m of w.d.

Terrain analyses on the obtained DTM were focused on quantitatively outlining the blocky pattern within which the coral mounds are densely distributed. A semi-automatic morphometric feature (i.e. seafloor geomorphology) extraction was performed over the computed TMAs in order to, as follows:

1. Outline the blocky pattern that is 600 km^2^ in area and that extends over the entire investigated sector of the northern Ionian Margin (Figure 6a). The RGB image (provided by the slope, the SD profile curvature, and the BPI index) represented in Figure 6a clearly enhances the blocky pattern of the investigated seafloor.
2. Isolate all of the mound-like features occurring within the study DTM (using the Unsupervised Classification). Each polygon represents a distinct feature of the entire DTM (Figure 2) with its own morphometric properties.
3. Model the coral-mound distribution through isolation of all of the mound-like features represented within the seismic profiles using a chaotic pattern exposed at the seafloor with very high acoustically transparent hyperboles [50] and within the depth range favourable to coral growth (i.e. from 400 to 1.000 m - [54]). We considered the polygons displayed in Figure 6b as those that best represent the morphometric properties of coral-mounds. Coral-mounds were determined to be 1,902 in total number with an average area of 35,000 m^2^ per mound, for a total of roughly 68 km^2^ (Figure 6b). Figure 6b provides information on how we modelled the coral-mound distribution on a mesoscale level.

Since we did not focus on a single species and since our goal was to predict the distribution of coral-mounds (i.e. the positive seafloor morphologies associated with the occurrence of CWC habitats), and not of a defined taxon, we did not apply a typical HSM to the available data. Specific geomorphometric techniques and geomorphological analysis were, instead, used to analyze DTM proprieties and to extrapolate the terrain features associated with coral-mounds. Our method was specifically designed for the study area, from which previous geomorphological and ecological studies allowed the identification of a “*geomorphological proxy*” associated with the coral mound distribution (Figure 6b).

In general, the application of predictive modelling in oceanic waters using mesoscale terrain parameters has revealed that seascape terrain attributes (i.e. TMAs) can be significant when predicting CWC habitat distributions [71], not just due to their direct influence on the coral distribution but because they likely serve as proxies for other processes. Also for our case, for example, the terrain orientation (i.e. aspect) most suitable for corals was due to the position relative to bottom currents that carried the food supply to the corals [72 and reference therein]. We also noted how, on the local scale, corals prefer elevated positions relative to the surrounding seabed that may be related to the nutrient supply and flow paths associated with mounds, as hypothesized [41] for our study area. Additionally, while a given species can be widely distributed (according to the results obtained from predictive modelling performed with HSM), a reef habitat or a coral-mound cannot, as discussed by [73] for *Lophelia pertusa* reefs. Not specifically an HSM, our habitat predictive modelling did not include water mass properties such as temperature and salinity that may also be crucial for defining the most suitable habitat for corals. We only selected, within the entire surveyed area, the bathymetric range (from 400 to 1,000 m of w.d.) where the ADW was detected from previous studies [55] and associated with coral occurrences [54].

Quantitatively, according to our results (Figure 6b), coral mounds alone make up a habitat equivalent in size to the average of the common Italian Marine Protected Area (MPA) included in the SPAMI (Specially Protected Areas of Mediterranean Importance) list (roughly 80 km^2^– [74]), although the total area with the dense aggregation of mound features (600 km^2^) is far more extensive than the area of coral mounds. The results provide instrumental information for conservationists, researchers, and governmental bodies. Maps representing the 600 km^2^ in which coral mounds are densely distributed (Figure 6) indeed represent a valuable tool for management programs of the SML CWC province. Spatial information regarding the habitat distribution reported in maps at the mesoscale level can support the definition of more precise regulations aimed at managing human use and activities within the marine environment.

### A Textural Analysis of Mound and Inter-mound Areas

All of the studied SSS-mosaics were located within the coral-mound areas modelled in Figure 6b. As reported for the MS04 and MS06 sites [41], seafloor roughness resulted in SSS mosaics within a very complex backscattering fabric that was enhanced by the presence of CWC facies. Computation of a supervised MLC algorithm on the six obtained SSS-textural bands, led to the identification of six acoustic classes on the mosaics (Table 2) from which we quantified coverage on the four SSS mosaics. The scatter plot in Figure 4 indicates that the selected six acoustic classes were quite well differentiated for their textural properties.

**Figure 7:**
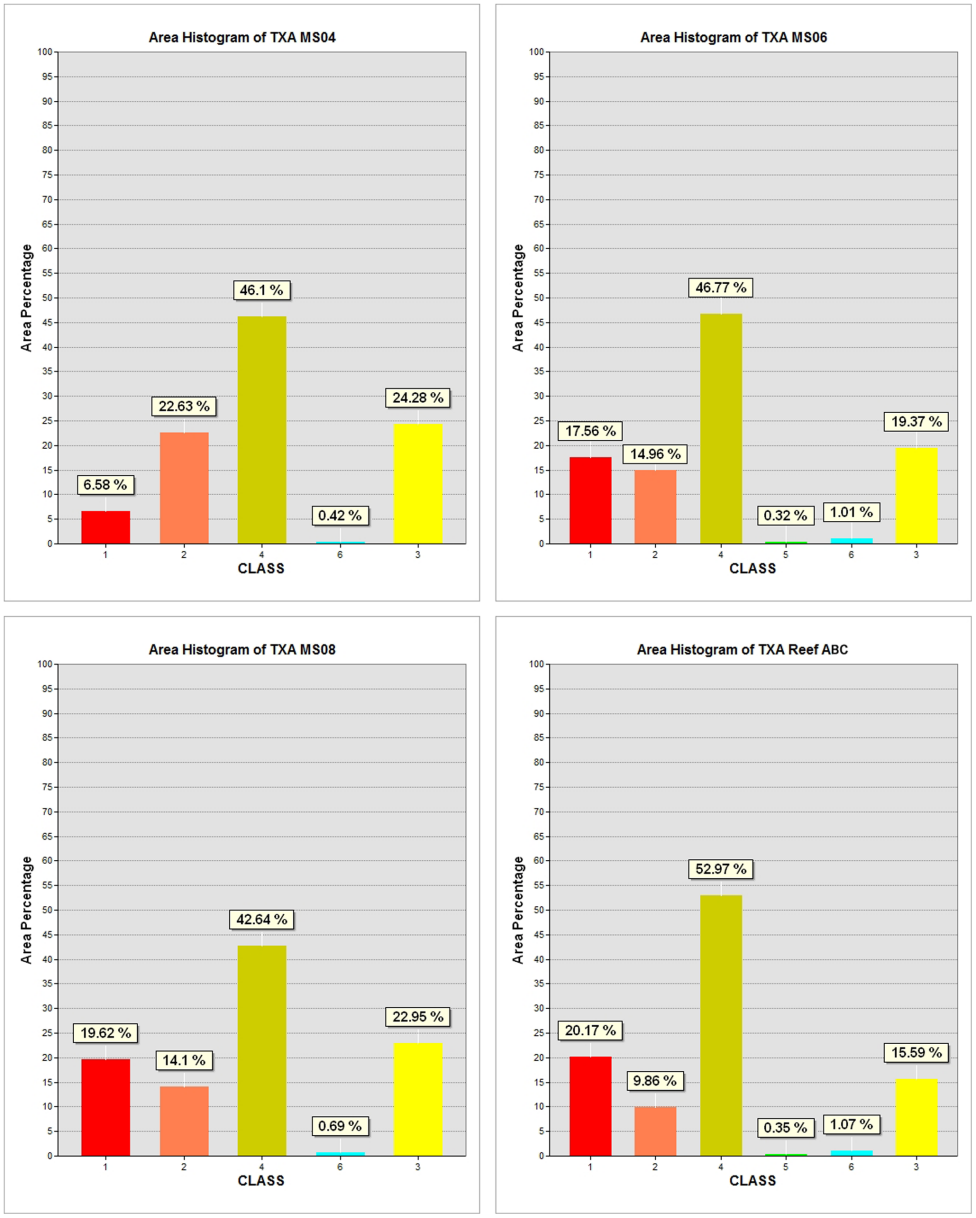
Graphs representing the classified results of the SSS mosaics shown in Figure 3. The maximum likelihood classification was performed on the six texture indices (i.e. image bands) calculated from the original images. The classification was based on six training classes that corresponded to the six acoustic facies listed in Table 3.

The percentage of coverage for acoustic classes for each mosaic is provided in Figure 7. Each class has a similar distribution range for the individual mosaics (Figure 7), consistent with our assumption that each mosaic was representative of similar seafloor properties (as also indicated from the sediment samples collected from the four sub-areas [61], [62]) that originated from the same sedimentary and geomorphic processes and that were colonized by the same typical benthic habitats (i.e. CWC).

The modelled distribution of coral mounds (Figure 6b) allowed us to extend the information produced from SSS textural analysis to the entire DTM (Figure 2). Indeed, the six acoustic classes actually represent the seafloor texture properties of the wider area of the DTM shaped by the blocky pattern with coral mounds (Figure 6a). As a result, identifying the sedimentary and biotic properties of each class allowed us to estimate the coverage percentage of sedimentary facies and biotic components for the entire 600 km^2^ where coral-mounds were densely distributed.

To determine the distribution pattern of the six acoustic classes between coral-mounds and inter-mound areas, we investigated the relationships between the mapped distribution of the six acoustic classes and the modelled distribution of coral-mounds (Figure 6b).

**Figure 8:**
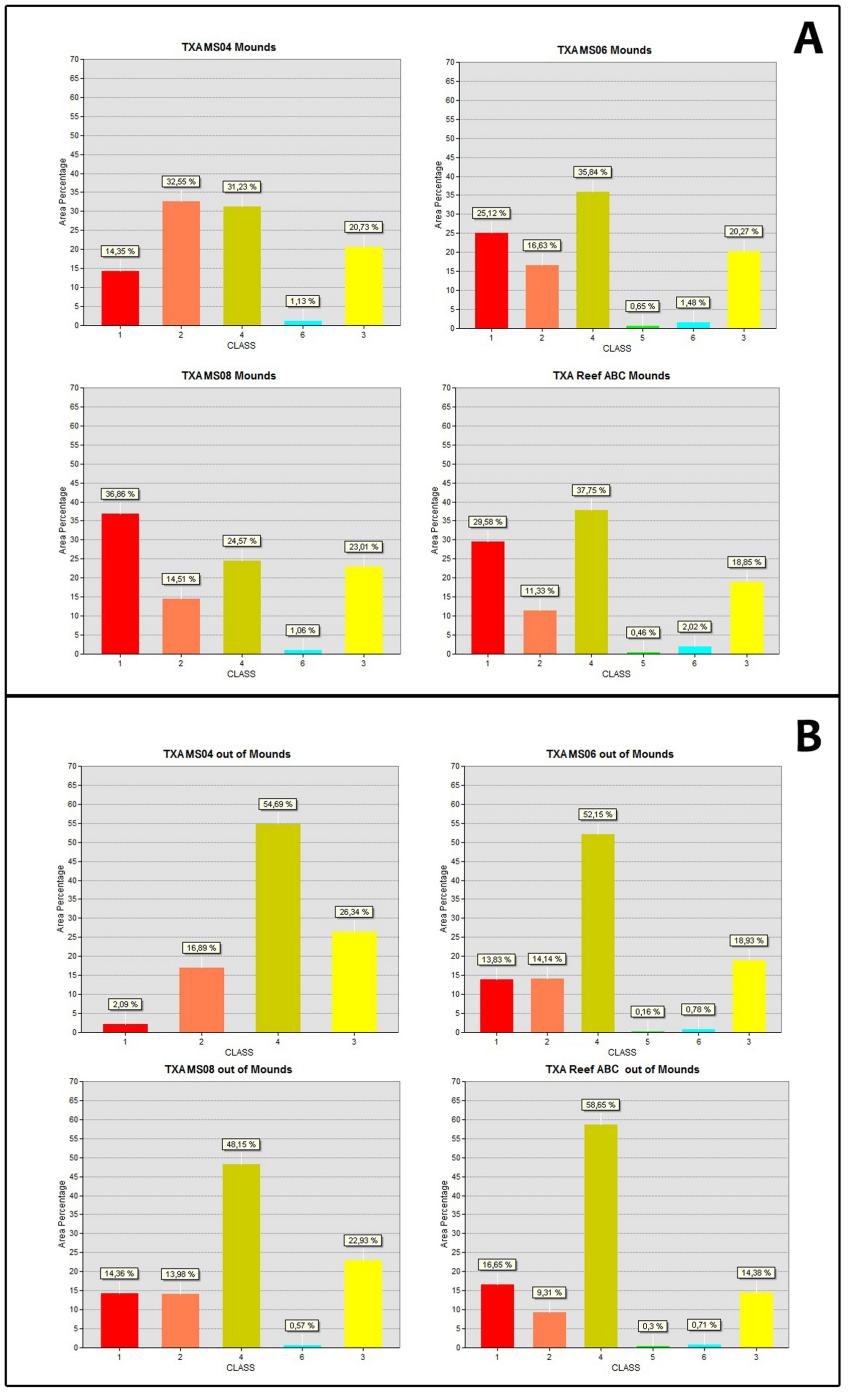
Graphs representing the classified results of the SSS mosaics (Figures 3 and 10) within (A) mound and (B) intermound areas.

**Figure 9:**
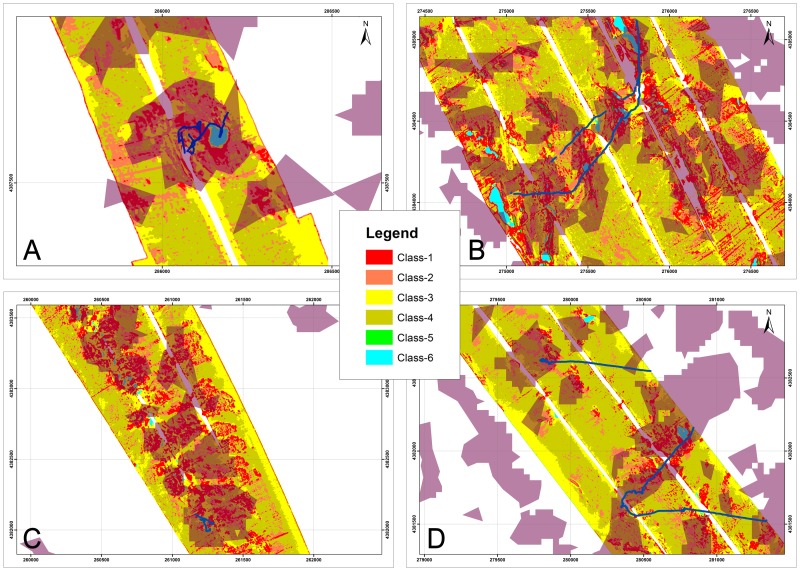
A: Details of the classified results of the SSS mosaic at sites (A) MS04, (B) MS06, (C) MS08, and (D) ReefABC. See Figures 2 and 3 for location. In A, B, and C the blue line indicates the MGS video survey tracks and the polygons in violet indicate boundaries of mound areas. In D the blue line indicates the QUEST4000 ROV-video survey track and the polygons in violet indicate the boundaries of mound areas.

Among the classes, Class 1 was definitely more abundant in mound areas than in inter-mound areas. (Figure 8). Class 2 was also preferentially distributed within mound areas (Figures 8a), although for some cases it was quite well-represented within inter-mound areas (Figures 8b) with similar percentage values (especially at the MS06, MS08, and ReefA sites). Observing their distributional pattern, Classes 1 and 2 were often strictly associated within their spatial extension and close to one another (Figure 9). Class 3 was, instead, well represented both in mound and inter-mound areas, while Class 4 was definitely more abundant in inter-mound areas (Figure 9). Classes 5 and 6 were poorly represented in all SSS mosaics and had the lowest values for percentages (<3%), and were not present in all of the mosaics (Figure 8). Their texture properties were selected by choosing the highest and lowest backscattering values (Table 1). The results indicate that Class 5 was only present within MS06 and ReefABC sites, both in mound and inter-mound areas, although more abundant in mound areas. Class 6 was only absent in the inter-mound areas of the MS04 mosaic. By observing their distributional pattern, we determined that Classes 5 and 6 were both better represented within mound areas or directly bordered these areas (Figures 8 and 9).

To better understand the real nature of the six acoustic classes, we investigated the correlation between the seafloor distributions of the six classes using results obtained from video analyses (i.e.: ground-truthing), as discussed hereafter following the presentation of results obtained from the video analysis.

### The Macrohabitat Characterization (Video Analysis)

Thirteen macrohabitats were identified on the basis of the % coverage of the following five dominant seafloor features: 1) coral colonies, 2) coral rudstones, 3) mudstones, 4) coral rubble, and 5) mud (Table 3). These features were recognized through video analyses and, in previous work, groundtruthed by sediment sampling [41], [61]. The features can be described as follows: 1) “coral colonies” - both dead and live colonies of the scleractinian species *Madrepora oculata* and *Lophelia pertusa*, exceptionally *D. cornigera* (they can be isolated (i.e. single colonies on soft or hard substrates) or aggregated to form skeletal frameworks (sensu Riding [75])); 2) “coral rudstones” and 3) “mudstones” - rocky substrates, mostly hardgrounds (see a detailed description in [41] and [61]); the former are limestones displaying a typically rough surface, obviously made up of fragmented coral branches and secondary cemented fine sediment; mudstones are herein identified as rocky substrates with a fairly regular and smooth surface, dominated by cemented silt to silty clay; 4) “coral rubble” - consists of the accumulation of loose, broken-off portions of scleractinian coral skeletons; and 5) “mud” - following the European Environment Agency Glossary (EEA 2011), is fine-grained sediment that includes silt and clay.

**Figure 10:**
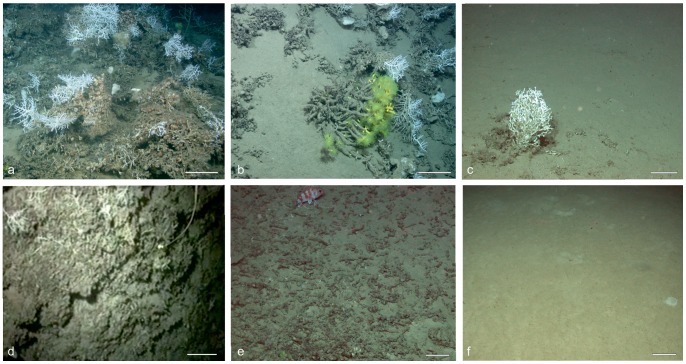
Some examples of video-detected macrohabitats. a: Coral framework (C) habitat with live *Madrepora oculata*, *Lophelia pertusa,* and *Desmophyllum dianthus*, Reef ABC site, © MARUM, Bremen University 2006. b: Coral and Mud (CM) habitat; note the yellow colony of *Dendrophyllia cornigera*, Reef ABC site, © MARUM, Bremen University 2006. c: Mud and Coral (MC) habitat with a single live colony of *L. pertusa*, Reef ABC site, © MARUM, Bremen University 2006. d: Coral rudstone (Crd) habitat with very sparse white *Madrepora* fragments (upper left), MS06 site. e: Coral rubble (Cr) habitat characterized by abundant dark coated scleractinian fragments; note the fish *Helicolenus dactylopterus* on the upper part of the image, Reef ABC site, © MARUM, Bremen University 2006. f: Mud (M) habitat showing some bioturbation, Reef ABC site, © MARUM, Bremen University 2006. Scale bar: a–d, 20 cm; e, 5 cm; f, 10 cm.

Among the 13 video-detected habitats listed in Table 3 and represented in Figure 10a–f, three are dominated by a relatively continuous and stable hard/lithified seafloor (C: Coral Framework, Figure 10a; Crd: Coral rudstone, Figure 10d; Ms: Mudstone), two are dominated by loose coarse and fine bio- and/or siliciclastic sediment (Cr: Coral rubble, Figure 10e; M: Mud, Figure 10f), and eight are characterized by transitional features (CM, Figure 10b; CrdM; MsM; CrM; MC; MCrd; MMs; and MCr).

The **C** (Coral framework) macrohabitat (Figure 10d) consists of laterally continuous aggregations of branched scleractinian coral colonies (primarily *Madrepora oculata* and secondarily *Lophelia pertusa*), whose skeletons are in mutual contact with the exception of some small sediment pockets (up to 0.25 m^2^). The coral framework is dominated by dead, and often black-coated colonies in the life position. Generally, the percentage of live colonies does not exceed 30%. More details regarding the fauna and the main sedimentological features of this coral-dominated habitat are found in [41] and [61]. The C macrohabitat was only recorded on the top (or on the upper north-eastern flank) of the examined mounds.

The seafloor of the **Crd** (Coral rudstone) macrohabitat (Figure 10d) is dominated (>75% per square meter) by a very rough, black-coated, rocky substrate consisting of coral rudstones and/or framestones made up of dominant fossil scleractinians and a subordinate mudstone to wackstone matrix [61]. Their surface may be locally colonized by very sparse live colonies (<25% coverage) and large white sponges. Crd macrohabitats are generally exposed on the top and upper flanks of topographic highs, and, seldom, on mid-slope scarps up to 1 m in height.

The **Ms** (Mudstone) macrohabitat is dominated (>75% per square meter) by a rocky seafloor (mudstone) with a fairly smooth surface, only locally irregular and crumble-like. Smooth hardground crusts were mainly observed on sub-horizontal and sub-vertical substrates (1–2 m high scarps) or on boulders derived from their dismantlement. Horizontal surfaces can partly be covered by a very thin layer of mud and are almost completely devoid of sessile macrofauna (Figure 10d). For more details see the description of the “Hardground macrohabitat” in [41] and [61].

The typical **Cr** (Coral rubble) habitat seafloor, scarce within the SML area (Table S2), is almost entirely covered by oxidized, bio-eroded, and heavily fouled colonial coral fragments only partly coated with a sprinkling of mud (Figure 10e). However, within this habitat we also included the dense “buried rubble” (sensu Vertino et al. [41]) that consists of closely packed coarse elements (from a few cm to several dm in size), apparently made up of coral fragments and/or coral rudstone slabs almost entirely covered by a thin layer of muddy sediment. The seafloor of the **M** (Mud) habitat (Figure 10f) is dominated (c. 100% per square meter) by moderately to strongly bioturbated silt to silty clay sediment. Dwellings, resting traces, and crawling trails (sensu Dundas and Przeslawski [76]) have commonly been observed. Additionally, in places, pennatulaceans such as *Funiculina quadangularis* and *Kophobelemnon stelliferum* can be frequent [77].

As shown in Table 3, the remaining habitats (CM, Figure 10b; CrdM; MsM; CrM; MC Figure 10c; MCrd; MMs, and MCr) are characterized by a variable percentage of the above-described main components (coral colonies, coral rudstone, coral rubble, mudstone, and mud). The occurrence of “Antipatharian Facies” (as described by [41] and [61]; Figure 7b-c in [41]), consistently characterized by meter-sized specimens of the black coral species *Leiopathes glaberrima* on a mixed seafloor are formed by hardground partly covered by mud or by mingled mud and boulders (mostly MsM and CrdM; Table 3).

### The Sea Truthing of SSS Mosaics

Georeferentiation of MGS-video tracks and SSS mosaics was not performed using the USBL system (see data and methods). Therefore, although the data are able to resolve macro-scale seafloor features, their accuracy in terms of the associated geographical position is not of the same order of magnitude for spatial resolution. In general, the use of sea-truthing is mandatory for SSS data interpretations, but often the collection of video data or sediment samples is not associated with proper data georeferentiation (e.g. by the use of accurate underwater positioning systems such a USBL), especially for the deep-sea environment. So, for our case, SSS data interpretation depended on the capacity to analyze seabed variability (investigated using sediment samples and/or video surveys) and to associate seabed variability to the variability in acoustic properties revealed by various backscattering textures. We initially adopted this approach to a comparison of the backscattering fabric of SSS mosaics to the distribution of the different seafloor macro-habitats defined through video-analyses (the same approach was adopted in [41]). To associate the six acoustic facies to the 13 macrohabitats, the acoustic facies must include more than one video-detected macrohabitat. According to the textural properties of the six classes (Figure 4) and their abundance and distribution in mound and inter-mound areas (Figures 8 and 9), we determined the following: (1) Class 1 has high backscattering values and a moderate variance in texture with high entropy and low homogeneity indices (Table 1 and Figure 5). Hard substrates and associated seafloor roughness generally produce such acoustic fabric in SSS data. According to the different macrohabitats detected from video analysis, the coral frameworks/rudstones and colonies distributed along the flank and/or on the top of coral mounds, represent the dominant portion of the seafloor resolved by SSS imagery able to produce such an acoustic response. The roughness of those habitats indeed provides an irregular pattern regarding a number of dark gray levels producing textures with high entropy and low homogeneity values [67]. Therefore, we considered C, Crd, Cm e CrdM (i.e. coral dominated macro-habitats) strictly associated to occur in Class 1. Since the resolution capacity of SSS instrumentation is not able to distinctly resolve hardground crusts when seafloor roughness is impacted by the occurrence of coral framework/rudstones and colonies, Ms and MsM can also be included in Class 1, especially when they are located within mound areas close to C, Crd, CM, and CrdM macrohabitats.

(2) Class 2 is formed from a larger number of moderate to dark gray levels than Class 1, which are arranged in unorganized patches that have quite a high variance, a low homogeneity, a high dissimilarity, and a high entropy (Figure 4). Class 2 has a less marked entropy than Class 1 and is distributed more within the mosaics, although preferentially distributed in mound areas, and also quite represented in inter-mound areas. Coral patchiness was indeed detected during the ROV-video analyses that provided a total of six different macrohabitats with the occurrence of coral colonies (C, Crd, CM, CrdM, MC, MCrd - Table 2). Among them, C, CM, Crd, and CrdM were considered representative of Class 1. Therefore, MC and MCrd should represent Class 2 that has textural properties similar to Class 1, but with less marked entropy formed by intermediate to high backscattering values as a result of the increased amount of sediment that attenuates seafloor roughness and backscattering intensity. Nevertheless, MC and MCrd can generate higher backscattering if they are distributed along the sloping seafloor. Therefore, especially in mound areas, MC and MCrd can easily produce the textural properties associated with Class 1.

(3) Class 3 has a lower backscattering intensity and a higher homogeneity than Class 1 and 2, and is well distributed both in mound and inter-mound areas. We associated this class with macrohabitats dominated by fine-grained sediment, often easily segmented in the textural analysis used for the SSS data classification, due to low variance and entropy and a high level of homogeneity (Figure 5). Class 3 should be representative of macrohabitats M, MCr, and MMs where the absence of coral colonies lessens the production of the roughness that can produce high backscattering intensity. Nevertheless, in case such habitats are located within mound areas and/or represent small sediment pockets within coral-dominated macrohabitats on the sloping seafloor (i.e. on the top or on the flank of the mound) they cannot be distinctly detected from SSS instrumentation as areas with low backscattering values and high homogeneity.

(4) Class 4 has an intermediate backscattering intensity, a low homogeneity, and a medium level of entropy; and the scatter in variance has a certain mix with coral facies. We associated this class to coarse sediments that are, indeed, difficult to quantitatively detect [60]. Within the studied area they consisted of the dominant occurrence of the following habitat components: (1) coral rubble, (2) coral rudstone fragments (from pebbles to boulders), (3) dismantled hardground crusts resulting in clustered to isolated elements highly colonized by solitary corals, and (more likely) by a mosaic of these components and mud. According to the various macrohabitats detected from video observations and the results obtained from the sediment sample analyses, we considered Class 4 as representative of Cr and CrM macrohabitats, although it was often difficult to distinguish M from MCr through the video analyses (the sedimentary grain proprieties were obviously not identified from video observations). Therefore, MCr can also easily produce a similar texture in SSS data.

(5) Class 5 has the highest backscattering values and is poorly represented in the SSS mosaics. When present it was localized on sub-vertical flanks. We considered Class 5 as representative of macrohabitat Ms and MsM, especially when they dominated the south-western flanks of the coral-mounds as described in [41] and when they represented the dominant portion of the seafloor.

(6) Class 6 is also poorly represented in SSS mosaics and has the lowest backscattering values created by shadow zones. Therefore, there was no correspondence with macrohabitats.

The DTM and SSS classification map overlay (Figure 9) also highlighted the sharp boundaries that the mounds revealed toward the east, often imaged by a well-defined limit between Class 1 or 2 extended within mound areas, and Class 3 or 4 falling in inter-mound areas, while the western mound flank was characterized by a gradational pattern (made up by the confused mix of different acoustic classes). Since the video analyses confirmed (as also as indicated in [41]) that the surface of the eastern mound flank was generally composed of coral facies, distinctly separated from the background sediment of inter-mound areas, we assumed that Classes 1 and 2 actually corresponded to the main coral-facies observed in video-analyses, consistent with the dominant distribution of Class 1 within mound areas (Figure 8).

To assess the accuracy of the computed SSS classification maps, we followed Pontius and Millones [69]. The results of the accuracy assessment revealed overall classification accuracy levels of 85% (Figure S1, Table S1 and text S1), and according to our interpretation, Classes 1 and 2 were considered as representative classes for coral dominated macrohabitats. For each mosaic, the percentage of coral facies represented roughly one third of the total area, with the highest percentage in MS08 (33, 62%) and the lowest for MS04 (29, 21) (Figure 7).

### Anthropogenic Impact and the Vulnerability of the CWC within the Northern Ionian Sea

**Figure 11:**
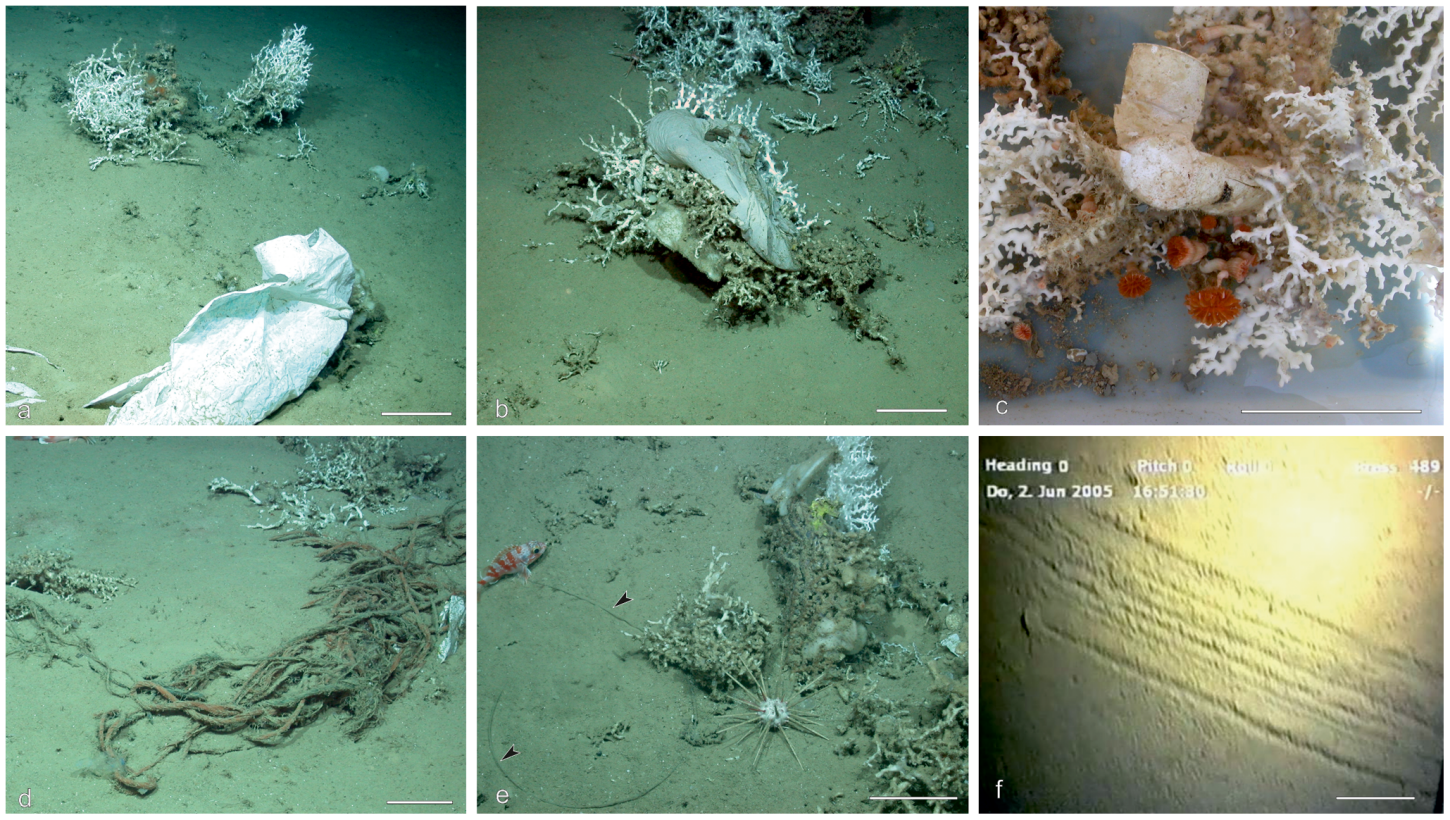
Human impacts on the seafloor. A–B: Plastic bags become entangled on coral colonies of MC habitats; Reef ABC site, © MARUM, Bremen University, 2006. C: Sample from the SML coral seafloor (73, CORSARO Cruise) showing a plastic glass overgrown by hydrozoans, a large colony of *M. oculata*, and several coralla of *D. dianthus*. D–E: Fishing longlines (blck arrows) and nets, respectively, among coral colonies from CM habitats; Reef ABC site, © MARUM, Bremen University, 2006. F: Trawling trace on the muddy seafloor (M habitat), MS06. Scale bar 10 cm.

**Figure 12:**
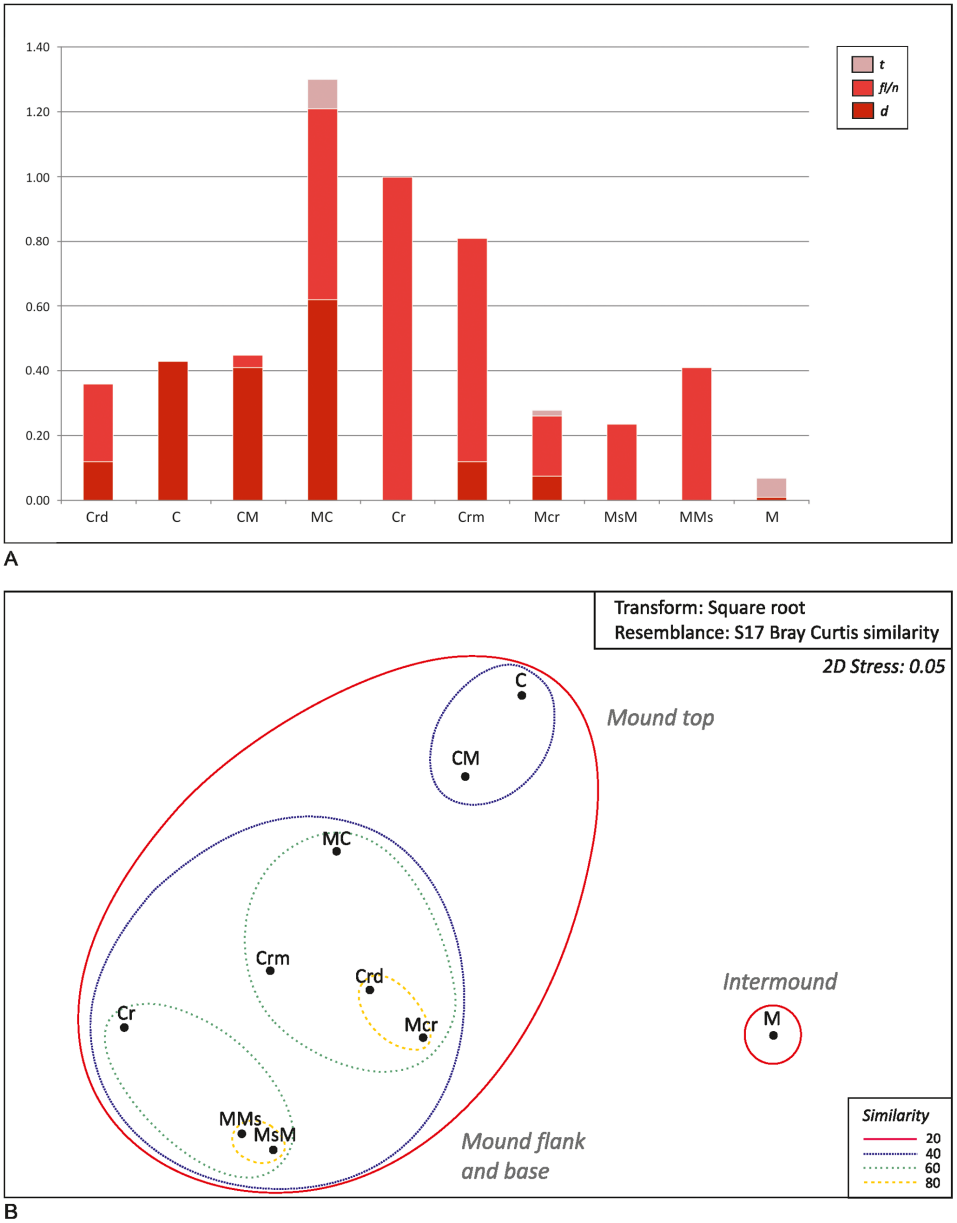
a. Histograms showing the abundance of anthropogenic items and traces (*d:* disposal, i.e. litter and solid waste; *fl/n*: rests of fishing lines and nets; *t*: trawling traces), identified through video analyses and expressed as the number of occurrences/10 m^2^, considering the total visualized area per each habitat. b. 2-dimensional MDS ordination of macrohabitats, as expressed in the last row of Table 4 (all sites), with superimposed clusters at similarity levels of 20%, 40%, 60%, and 80%.

During the video survey, several anthropogenic impacts were detected and recorded following the classification proposed by [3]. We distinguished the following two main categories: 1) disposal (litter and solid waste), and 2) fishing exploitation (trawling traces and the rests of fishing lines and nets). The observed litter primarily consisted of plastic material (bags, sheets, containers, bottles, and glass; Figure 11a–c), and, secondarily, metal, glass, and cloth. Litter was observed at all of the examined sites and was especially associated with coral habitats (mostly C, CM, and MC, Table 4; Figure 12a); in particular, plastic bags were found to be exclusively entangled in live and dead coral branches (Figure 11a–b). Due to the difficulty in distinguishing them among densely-packed black and white scleractinian branches, our count of white and black plastic items is likely underestimated in coral-dominated habitats. Lost or discarded fishing lines (related to longline fishing) and subordinate nets were mainly recorded in association with coral rubble (e.g. Cr, CrM habitats), boulder fields (e.g. MMs, MsM habitat), and loosely packed coral habitats (MC) (Table 4, Figure 12a) typically entangled on coral branches/fragments (Figure 11d–e) and boulders. Well marked trawling traces (Figure 11f) were exclusively identified within mud-dominated sediments (M, MCr, and MC; Table 4) in the vicinity or at the base of coral mounds or in between coral mounds.

**Figure 13:**
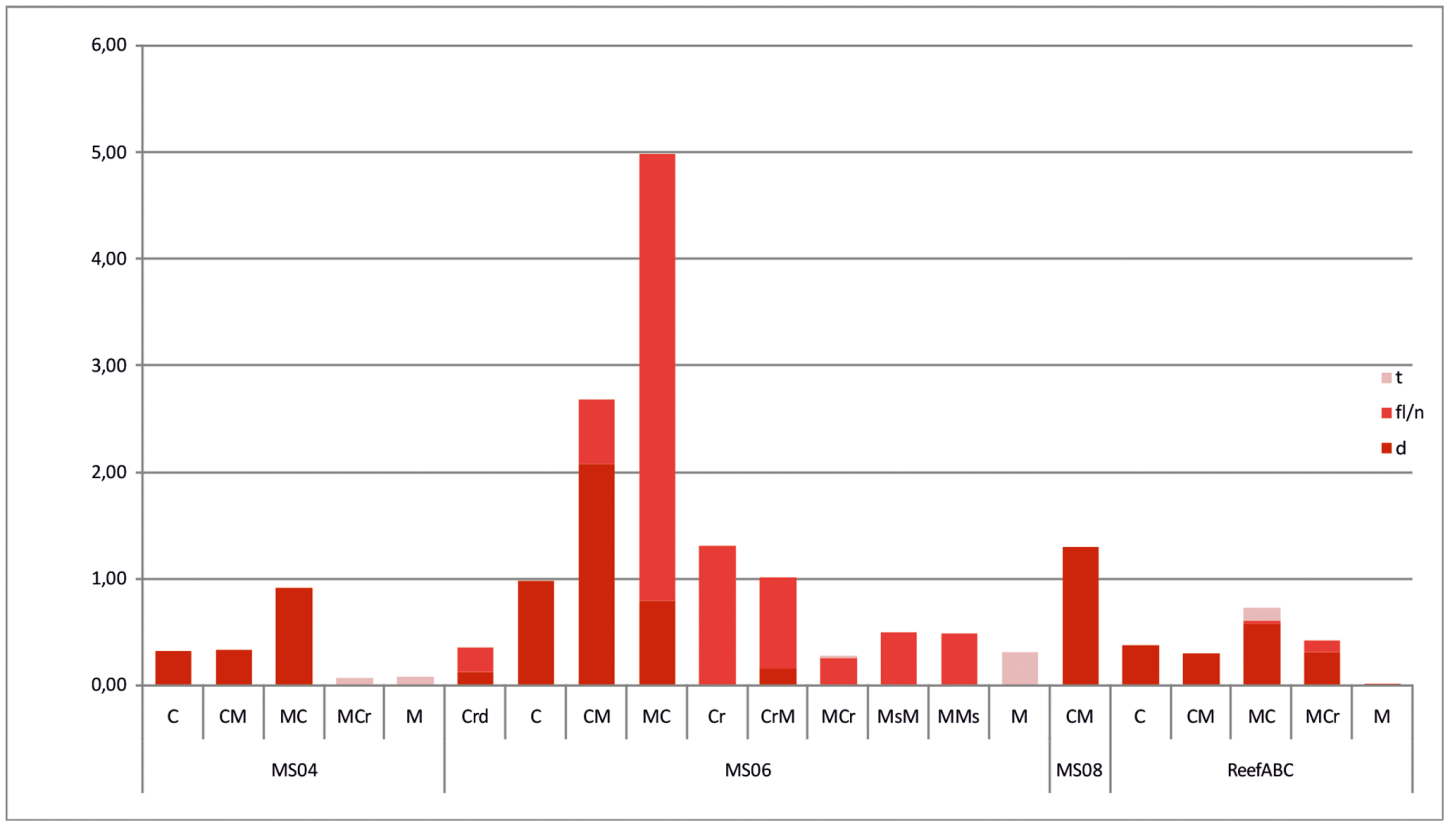
Histograms showing the abundance of anthropogenic items and traces (*d:* disposal (i.e. litter and solid waste); *fl/n*: rests of fishing lines and nets; *t*: trawling traces), identified through video analyses and expressed as the number of occurrences/10 m^2^, per habitat and per site.

Anthropogenic impact is much more evident at MS06 than at the other sites (Table 4 and Figure 13), both for disposal and fishing exploitation. Additionally, the results of the cluster and MDS analyses (Figure 12b) indicate that similar habitats, in terms of both biological/sedimentological composition and location with respect to morphological features, are subject to similar anthropogenic impacts. In particular, the macrohabitat MDS ordination related to anthropogenic impact (Figure 12b) indicates a clear differentiation between bioturbated mud habitats, mostly developed within intermound areas [41], and coral/boulder-related habitats that are typical of mound tops and flanks. Within mound habitats, two additional clusters were determined. The first has an internal Bray Curtis average similarity of approximately 86% (see the SIMPER results in Table S3) characterized by coral-dominated habitats (C and CM) typically located on the mound upper portion [41]. The second, more heterogeneous and showing an internal similarity of approximately 65%, grouped the habitats preferentially located at the base of the mound flanks (MC, Cr, Crm, Mcr, MMs, etc.) [41]. A clear differentiation also exists between coral habitats typically located on the upper (C-CM) and lower (MC) portion of the mound eastern flanks and the boulder field habitats (MsM and MMs) that prevail on the mound western flanks [41]. The two clusters, C-CM and MsM-MMs, display an average dissimilarity of approximately 86%, as explained by both solid waste (dominating the former group; Table 4, Table S3, Figure 12a) and fishing line or net rests (more common for the latter group; Table 4, Table S2, Figure 12a); whereas the differences (∼54% dissimilarity) between CM and MsM-MMs habitats are largely explained by solid disposal (Table S3). The Simper Test also revealed that the distinction (of approximately 81% B–C dissimilarity) between the intermound M macrohabitat and the top-mound coral habitats is mostly explained, by approximately 62%, by the abundance of solid waste (mainly plastic bags, very common in C and CM habitats and almost absent in M). The high abundance of plastic bags in coral-dominated habitats seems to be related to the “trapping effect” that branching colonial corals, located on geomorphological highs and therefore exposed at strong current speeds, can exert on light material moved by bottom currents. Indeed, it has often been reported that coral-mounds develop through coral-growth and consequent sediment trapping during the time of accretion [71].

Within the Leuca area, most of the light plastic material identified in the videos and entangled in the coral branches was likely dumped by ships and boats, but could have also arrived from the coast. Due to the south-westerly main bottom current [54], light plastic material can easily be transported within the water mass and, bumping against the eastern mound flanks, become entangled in coral branches. In some cases, litter has even been found to be incorporated into the skeletons of living coral colonies (Figure 12c). Very little is known regarding the impact that plastic and other litter materials have on benthic organism physiology [78]. Since it may cause benthic fauna suffocation and, in case of plastic bottles and containers, can be associated with the release of toxic chemicals [79], intuitively, a high amount of plastic material on the seafloor is considered to be a serious threat for the health of CWC communities.

In the study area, coral habitats have been reported to play an important role as an attraction-refuge for fish, with respect to neighbouring barren muddy bottoms where fishing activity is more severe [57], [80]. Hard substrates interspersed with coral mounds and muddy bottoms are less accessible to fishing activities, and, therefore, can provide a natural refuge for mobile fauna, as observed in submarine canyons [81]. In this regard, D’Onghia et al. [80] detected greater abundances and sizes of fish within the Leuca area shaped by a blocky pattern, where fishing occurred only in some peripheral zones. Such an observation is consistent with the results obtained from our video analyses, which documented more records of fishing activity at the MS06 site located at the western limit of the blocky area (Figures 2 and 3) as compared to other areas (Table 4, Figure 13). Here a considerable amount of lost or discarded fishing lines and nets (up to four occurrences every 10 m within the MC habitat!) have been indistinctly recovered on both the eastern and western mound flanks, and, only exceptionally, on purely mud habitats where trawling marks have been documented. No evident trawl marks and very few fishing lines and nets were found, instead, in Reef ABC that is roughly located at the center of the blocky area (Figures 2 and 3; Table 4, Figure 13), implying that, at the meso-scale, the morphology of the coral mound province plays an important role in reducing the impact of trawl-fishing activity on CWC habitats.

Such observations could support and drive the establishment of selected areas to be protected, without excluding areas in which fishing activity can be performed without an important impact. In addition, the geomorphological interpretation supported by the production of the DTM (Figure 2) can identify areas in which bottom trawl activity altered the hydrodynamic and sedimentary regime, indirectly impacting coral habitats and associated benthic fauna [82].

The potential for managing human threats and facilitating the sustainable use of ocean resources relies on the delivery of reliable scientific information regarding the occurrence, distribution, and knowledge of the biology and ecology of deep-sea fauna to resource and policy managers. To address the management requirement, an urgent need exists for the development of robust methods for mapping marine ecosystems in order to establish their geographical location, extent, and condition. Such a process is only just beginning in the deep sea [83]. The improvement of habitat mapping methodologies for estimating the distribution and quantity of deep-water habitats, combined with additional information related to water masses (such as temperature, salinity, nutrient supply, current intensity, and direction) and human impacts, will enhance our ability to differentiate levels of habitat vulnerability according to the characterization of the biotic and abiotic components of a given habitat to better preserve deep-sea ecosystems. Our results indicate the high potential of acoustic surveys, if appropriately ground truthed, to not only map CWC coral habitat distributions but to also indirectly estimate the impact of certain types of human threats on the deep seafloor.

## Conclusions

The work presented here provides a realistic estimate of CWC-coverage within the study area and information at scales relevant for MSP and EBM. Our approach should offer an efficient and cost-effective technique for supporting the growing global need for better spatial management within the Mediterranean marine environment. In particular, our analysis reported that within the entire sector of the investigated margin (Figure 2–2,000 km^2^), 5,820 mound-like morphologies were isolated and were particularly aggregated over a total area of approximately 600 km^2^ (i.e. area of the blocky pattern). Furthermore, roughly 82% (in terms of occupied area), for a total of 1,902, could be coral mounds, for a total area of 68 km^2^.

An analysis of anthropogenic impacts indicated how seafloor geomorphology can influence habitat distribution and, therefore, human impact on benthic habitats. On the one hand, the blocky pattern in which SML coral mounds are distributed protects associated habitats from severe fishing activity. On the other hand, our data revealed severe impact on the CWC facies produced by waste material that was enhanced by the local scale morphological pattern. Since we documented how different habitats can have a different level of vulnerability to human pressures according to their structural nature and their relationship to seafloor geomorphology, our results suggest reviewing and better defining the concept of deep-sea habitat vulnerability.

## Supporting Information

Figure S1 **Results from the SSS classification map accuracy assessment (according to** [**68**] **.** (A): Commission, agreement and omission; (B): Allocation and quantity disagreements.
(TIF)

Table S1 **Cross-tabulation matrix of the SSS classification map at ReefABC site.**
(XLSX)

Table S2 **Matrix showing the examined area (expressed in m^2^) of the habitats impacted by human disturbance and the number of anthropogenic occurrences (garbage disposal (** ***d*** **), rests of fishing activity (** ***fl/n*** **); trawling traces (** ***t*** **)) identified through video analysis, per habitat and per site.**
(XLS)

Table S3 **SIMPER results indicating the following: (1) similarities within, and (2) dissimilarity between the habitat clusters discussed in the text and presented in** **Figure 13b** **; and (3) the contribution of the considered variables (human threats:** ***d, fl/n, t*** **) to those similarities/dissimilarities.**
(XLS)

Text S1 **Supplementary methods, results, and discussion for the accuracy assessment of SSS classification maps.**
(DOCX)
