# Correction: Mapping Cold-Water Coral Habitats at Different Scales within the Northern Ionian Sea (Central Mediterranean): An Assessment of Coral Coverage and Associated Vulnerability

**DOI:** 10.1371/journal.pone.0091447

**Published:** 2014-02-27

**Authors:** The PLOS ONE Staff

The affiliations for all authors are incorrect. The first three authors (Alessandra Savini, Agostina Vertino and Fabio Marchese) are affiliated only with institution number 1, Milano-Bicocca University, Dept. of Geological Sciences and Geotechnologies, Milano, Italy, and not with institution number 2, and the last two authors (Lydia Beuck and André Freiwald) are only affiliated with institution number 2, Senckenberg am Meer, Marine Research Department, Südstrand, Wilhelmshaven, Germany, and not with institution number 1.
