# Correction: Mapping Cold-Water Coral Habitats at Different Scales within the Northern Ionian Sea (Central Mediterranean): An Assessment of Coral Coverage and Associated Vulnerability

**DOI:** 10.1371/journal.pone.0102405

**Published:** 2014-07-03

**Authors:** The PLOS ONE Staff

There is an error in the affiliations for all authors. The previous corrections notice listed the incorrect department for Dr. Alessandra Savini, Dr. Agostina Vertino e Dr. Fabio Marchese. The correct affiliations and departments for all the authors are:

Alessandra Savini, Agostina Vertino and Fabio Marchese: Milano-Bicocca University, Department of Earth and Environmental Sciences, Milano, Italy,

Lydia Beuck and André Freiwald: Senckenberg am Meer, Marine Research Department, Südstrand, Wilhelmshaven, Germany.
