# Supplementary figures and images for: Mapping Cold-Water Coral Habitats at Different Scales within the Northern Ionian Sea (Central Mediterranean): An Assessment of Coral Coverage and Associated Vulnerability
Source: PLoS One. 2014 Jan 23;9(1):e87108. doi: 10.1371/journal.pone.0087108 (PMC3900717; doi:10.1371/journal.pone.0087108)

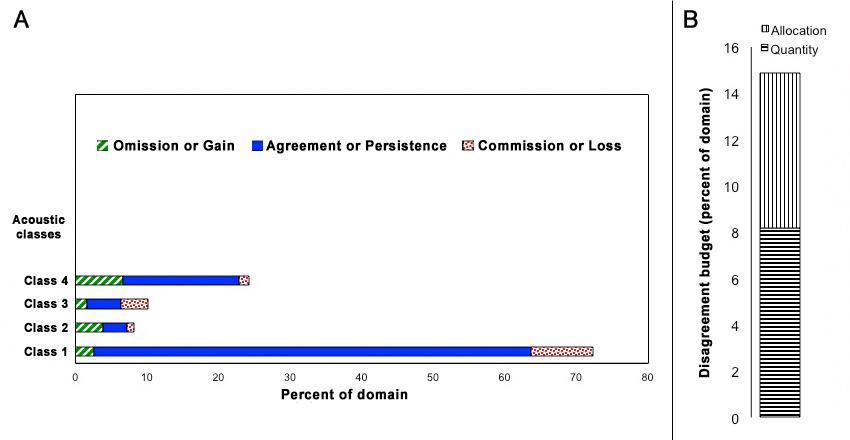

Supplement: Figure S1 — Results from the SSS classification map accuracy assessment (according to [68] . (A): Commission, agreement and omission; (B): Allocation and quantity disagreements. (TIF) [file pone.0087108.s001.tif]
