# Supplementary material for: Mapping Cold-Water Coral Habitats at Different Scales within the Northern Ionian Sea (Central Mediterranean): An Assessment of Coral Coverage and Associated Vulnerability
Source: PLoS One. 2014 Jan 23;9(1):e87108. doi: 10.1371/journal.pone.0087108 (PMC3900717; doi:10.1371/journal.pone.0087108)
Supplement: Text S1 — Supplementary methods, results, and discussion for the accuracy assessment of SSS classification maps. (DOCX) [file pone.0087108.s005.docx]

Text S1. Supplementary methods, results, and discussion for the accuracy assessment of SSS classification maps

Supplementary Methods: The Pontius matrix

Pontius and Millones [1] proposed a useful and simple approach for accuracy assessment that focuses on two components of disagreement between maps in terms of the quantity and spatial allocation of categories, namely *quantity disagreement* and *allocation disagreement*. Quantity disagreement is defined as ‘the amount of difference between the reference map and a comparison map that is due to the less than perfect match in the proportion of the categories’, whilst allocation disagreement is defined as ‘the amount of difference between the reference map and a comparison map that is due to less than the optimal match in the spatial allocation of the categories, given the proportions of the categories in the reference and comparison maps’ [1].

Pontius and Millenos [1] proposed this approach for accuracy assessments for a classified land use/cover map obtained using an analysis of remote sensing data in the terrestrial environment. Since the method is reasonably recommended, instead of Kappa indices, when the main purpose of the work focused on the quantification of cover of a particular class computed using a classification of remote sensing data [1], we adopted this approach for the accurracy assessment of remote data acquired in the submarine environment. Therefore, our SSS data represents the remote sensing data we analyzed in the submarine environment for quantifying the coverage of acoustic classes associated with coral occurrences. Reference information was obtained from video analysis results on straight segments of the ReefABC ROV-track (the most accurate georeferenced video data we had for the investigated area). Given the density of information obtained from the video analysis along the track (spaced by at least 1 meter), mapped video data (categorized using the macrohabitat classes defined in section 4.3) did not have the same spatial scale as the SSS classification map (1:5000). Therefore, to obtain the same density of information between the interpreted video data and the SSS classification map, the video data were decimated. In particular, we merged the interpreted video data into a grid with a 5 x 5 grid cell size (the same as the computed SSS classification map) and only the macrohabitat corresponding to the majority of observations in each cell was kept. The macrohabitat was then grouped and associated to four acoustic classes (classes 1, 2, 3, and 4), as described in section 4.4. An accuracy assessment for Classes 5 and 6 was not considered because the macrohabitat we associated with Class 5 (Ms) was not detected in the reference data we used, and there was no correspondence with the macrohabitats for Class 6. In addition, Classes 5 and 6 were both poorly represented within the SSS classification maps (the cover percentage was 0.35 for Class 5 and 1.07 for Class 6 at the ReefABC site).

**Supplementary Results and Discussion: Accurracy assessment**

The results of the accuracy assessment revealed overall classification accuracy levels of 85% (Table S1). Figure S1 presents the details of the allocation and quantity disagreements, including the commission, agreement, and omission for each acoustic class. Allocation disagreement always occurs in pairs of misallocated pixels [1]. A single pair consists of one pixel of omission for a particular acoustic class and one pixel of commission for the same class. An omission pixel is defined as a pixel for a particular class that appears on the reference map, but not on the comparison map (e.g. the pixel belongs to Class 1 in the reference data (i.e. video data), but not in the comparison map (i.e. SSS classification map)). A commission pixel is the opposite; where a pixel for a particular class appears on the comparison map and not on the reference map.

The results obtained through conversion of the sample matrix into a population matrix (according to [1]) indicated that, in terms of agreement, Class 1 (C, Crd, CM, CrdM) had the highest with 61%, while Class 2 had the lowest with 3%. Class 2 was, also, the only Class that had an agreement (3%) lower than the omission percentage (4%). As specified in section 4.4, Class 2, indeed, had textural properties similar to Class 1, especially in mound areas, where MC and MCrd could easily produce the textural properties associated with Class 1, at the expense of Class 2 (indeed, Class 2 also had the lowest commission of 1% and Class 1 had the highest commission of 9%). The exact distinction between Class 1 and Class 2 was actually difficult to obtain, nevertheless they both represent macrohabitats associated with coral occurrences; and our focus was to quantify the total coverage produced by both Classes.

Class 4 had the highest omissions, 7% (Figure S1), and the lowest commission, 1%, while Class 3 had relatively high commissions (4%) if compared with the agreement of 5%. For this case, we assumed that Cr and CrM macrohabitats (representative of Class 4 with MCr) can easily produce strong backscattering (similar to Classes 1 or 2), especially when determined in small sediment pockets located in mound areas or close to C and Crd macrohabitats; in this case Classes 1 or 2 (which represent the dominant habitats in mound areas) could gain cover percentage at the expense of Class 4. Class 3 (M) was, instead, not easy to find in mound areas where the production of coral rubble can be significant, affecting the sediment composition with a coarse carbonate sediment. According to the results from the video analysis, Class 3 dominates intermound areas, and the 4% of commission in Class 3 suggests that MCr can also easily produce a similar texture in SSS data, as stated in section 4.4.

In conclusion, the overall classification accuracy levels of 85% and the highest agreement obtained by Class 1 allowed us some degree of confidence in proceeding with the assessment of coral coverage using quantification of the entire area occupied by Classes 1 and 2.

**References:**

[1] Pontius RG, Millones M (2011) Death to Kappa: Birth of quantity disagreement and allocation disagreement for accuracy assessment. Int. J. Remote Sens. 32, 4407–4429.
